# Patient participation in gastrointestinal endoscopy — From patients' perspectives

**DOI:** 10.1111/hex.13066

**Published:** 2020-05-05

**Authors:** Hanna Dubois, Johan Creutzfeldt, Monita Törnqvist, Mia Bergenmar

**Affiliations:** ^1^ Department of Clinical Science, Intervention and Technology Karolinska Institutet Stockholm Sweden; ^2^ Karolinska University Hospital Stockholm Sweden; ^3^ Department of Oncology‐Pathology Karolinska Institutet Stockholm Sweden; ^4^ Sophiahemmet University Stockholm Sweden

**Keywords:** attitudes, colonoscopy, communication, endoscopy, gastroscopy, gastrointestinal, health knowledge, patient participation, patient‐centred care, practice, professional‐patient relations, qualitative research, sigmoidoscopy

## Abstract

**Background:**

Patient participation is associated with satisfaction and improved health‐related outcomes. In gastrointestinal endoscopy, patient participation is an underexplored area.

**Objective:**

To gain understanding on patients' experiences, attitudes and preferences concerning patient participation in the endoscopy pathway.

**Methods:**

Semi‐structured interviews with endoscopy patients (n = 17, female n = 8, male n = 9, ages 19‐80 years) were performed. Interview transcripts were analysed using qualitative content analysis. Participants were recruited by purposive sampling from an endoscopy unit in a Swedish university hospital. Inclusion:≥ 18 years, fluency in Swedish and recent experience of endoscopy at the unit.

**Results:**

Five generic categories emerged, two within the area of the patient's role, which was described as active or passive/included or excluded. Another three generic categories related to factors, critical to active participation, including organizational aspects, impressions of staff and individual circumstances were identified. In this context, patient participation described in the interviews was on a low to basic level, although sometimes reaching a higher level when staff ‘invited’ patients in decision making.

**Discussion:**

This study contributes to the understanding of patient participation in endoscopy. Patients are in an inferior position and need support from the staff for an active role in their care. Although there were variations on the perceived importance of different factors, a heavy responsibility lies on the endoscopy staff to acknowledge the patients' individual needs and to facilitate patient participation.

**Conclusions:**

Endoscopy staff has a key role in supporting patient participation. In endoscopy settings, patient participation is vulnerable to multiple factors.

## 1 INTRODUCTION

The Institute of Medicine advocates *patient‐centred care* as one of the core competencies for health‐care professionals, regardless of discipline.^1^ An approach where patients' individual needs and preferences are met, is increasingly recognized as a part of high‐quality modern‐day health care. Besides supporting patient autonomy,^2^ patient‐centred care has been associated with improved care experiences, self‐management and health‐related outcomes.^3^, ^4^, ^5^, ^6^

The scientific literature on patient‐centred care is ambiguous in terminology and definitions. Patient participation, patient‐centred care and patient engagement are examples of related concepts. Common denominators include the following: well‐informed patients, care in respect to patients' preferences, and a strive for involvement of patients in planning of care and decision making.^7^, ^8^, ^9^, ^10^ Several researchers point out that these related concepts are often used synonymously in the literature, although with slight differences.^7^, ^8^, ^11^ In this paper, we explore *patient participation*, identified as the basis for patient‐centred care,^8^ in the context of gastrointestinal (GI) endoscopy.

In a concept analysis by Cahill, defining attributes of patient participation include the following: a relationship between the patient and the professional where the patient's individuality is respected, narrowing of the information‐ and knowledge gap between them, willingness on the part of the health‐care professionals to shift power to the patient, and on the patient's part to assume a degree of power, control and responsibility.^11^ We have been guided by Cahill's description of patient participation in this study.

Carman et al proposed a multi‐dimensional framework, where patient engagement occurs on three levels: (a) direct care, (b) organizational design and governance and (c) policymaking. The authors also showed that patient engagement can occur in varying degrees. On the level of direct care *being informed* comprises the most basic form of engagement. When patients are *asked about their preferences*, patient engagement reaches a more developed form, and finally, when *decisions are made based on the patient's preferences, medical evidence and clinical judgement*, a type of partnership between the patient and the professional is established.^7^ In this paper, we have used the degrees of engagement in Carman et al's framework to assess patient participation.

Gastrointestinal endoscopy includes several common examinations, such as gastroscopy, sigmoidoscopy and colonoscopy. These can be performed under general anaesthesia, but in many countries and hospitals, conscious sedation^12^ or no sedation is standard routine. Anxiety associated with endoscopic procedures has been reported in many studies.^13^ Most people undergoing GI endoscopy are outpatients. In this study, the *GI endoscopy pathway* means the process from a decision being made about investigation to the results being communicated.

Although patient‐centredness is considered a quality indicator in GI endoscopy,^14^ there is limited scientific literature on the topic. Patient experiences have been studied to understand and improve adherence to colon cancer screening programs.^15^, ^16^, ^17^ There are some studies that have explored patient experiences and patient‐centredness as a quality measure in GI endoscopy. However, in these studies, questionnaire‐based instruments have often been used.^18^ As pointed out by several authors, many of the questionnaires have not been developed from actual patient narratives, but from the clinicians' or researchers' point of view, and therefore do not necessarily reflect the patients' perspectives.^18^, ^19^ Indeed, clinicians do not always estimate patients' preferences accurately.^20^, ^21^ In a study where endoscopists rated items they thought were of importance to patients, the importance of physical comfort was overestimated and that of shared decision making and information was underestimated.^20^

There is a lack of validated patient‐derived endoscopy experience measures.^18^ An understanding of what patients value in their endoscopy experience is crucial for developing patient‐centred endoscopy care.^22^

In studies where endoscopy patient experiences have been examined, the reports include descriptions of aspects important for patients, that are highly relevant for the patient‐centred approach, for example the importance of the trustful patient‐professional relationship, sense of control over one's condition and need of information.^23^, ^24^, ^25^, ^26^ However, the aims of previous research in the endoscopy field have not included exploration of patient‐centredness per se. Thus, to understand what patient participation means in the context of GI endoscopy, patient narratives focused on patient participation are needed.

The aim of this study was to gain understanding on patients' experiences, attitudes and preferences concerning patient participation in the GI endoscopy pathway. The research questions were the following:

1. How do patients in GI endoscopy describe their experiences, attitudes and preferences on patient participation?
2. What obstacles and facilitators to patient participation in GI endoscopy can be identified?

## 2 METHODS

### 2.1 Context

The setting of the study was an endoscopy unit at a university hospital in Huddinge, Sweden (Karolinska University Hospital). At the time of the study, approximately 4000 endoscopies were conducted yearly, the majority being elective procedures.

At the unit, most patients are examined either unsedated or by conscious sedation (midazolam and/or alfentanil). A small number of patients have access to non‐anaesthesiologist propofol sedation, while endoscopy requiring general anaesthesia is performed at the hospital's operation unit. Procedures performed at the endoscopy unit are diagnostic gastroscopy (including endoscopic ultrasonography), sigmoidoscopy and colonoscopy, as well as therapeutic procedures such as polypectomy and endoscopic submucosal dissections. A checklist combining patient safety and person‐centred care is used at the unit.^27^

### 2.2 Sampling strategy

Study participants were recruited by purposive typical case sampling,^28^ that is striving to find participants differing in age, gender and cultural background, as close to the typical patient demography at the unit as possible. Inclusion criteria were the following: outpatients who had undergone endoscopy at the study unit, ≥18 years of age and fluency in Swedish. Exclusion criteria were the following: procedure in general anaesthesia, involvement of any of the researchers during the endoscopy and participation in other research projects at the unit. Two research nurses at the unit, not in other ways involved in this study, identified eligible patients and approached them with written information. Patients who were interested in participating consented to their contact information being forwarded to the interviewer, who a few days later contacted them by telephone to schedule an in‐person one‐to‐one interview at the hospital, in a room, not located at the endoscopy unit.

The recruitment of study participants started in October 2017. The research group assessed data saturation after 14 interviews. However, scheduling interviews with younger adults (<40 years) was difficult, possibly due to their work and family engagements; thus, data collection continued until November 2018 to include younger adults. The research group reassessed data saturation and discontinued data collection at 17 interviews.

### 2.3 Data collection methods

**FIGURE 1:**
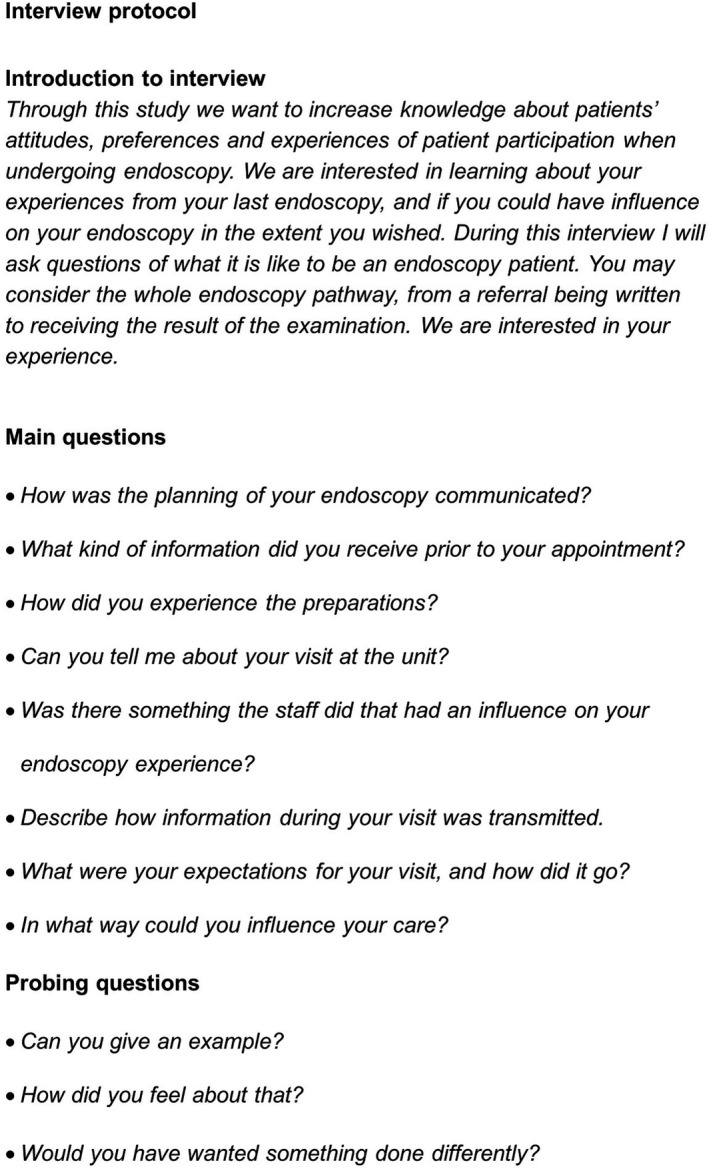
Semi‐structured interview protocol

A semi‐structured interview guide was developed within the research group (Figure 1). To avoid participants being limited by pre‐conceptions, a definition of patient participation was not provided during the interviews. Questions in the interview guide were broad, allowing participants to freely discuss their thoughts, preferences and experiences of participation. Probing questions guided the interviews on aspects brought up by the participants that could be related to patient participation, as defined by Cahill.^11^ To assess face and content validity of the interview guide, it was used in a pilot interview. After this, one question in the interview guide was slightly revised for clarification. As revisions of the interview guide were minor, the pilot interview was included in the study. As a gesture of appreciation for their invested time, the participants received a gift card at the cinema, with the value equivalent of one movie ticket.

**FIGURE 2:**
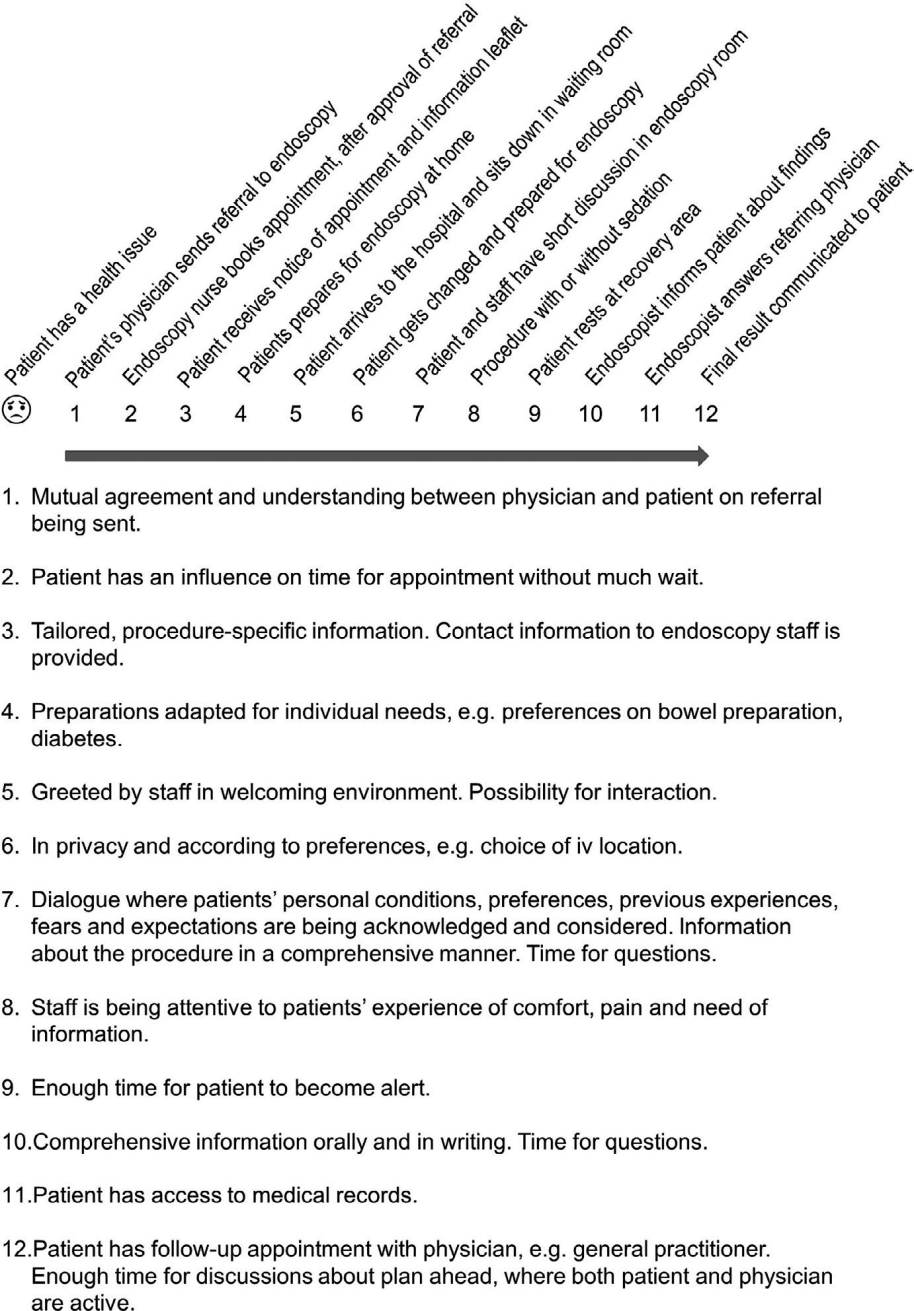
The endoscopy pathway with examples of patient participation, derived from the interviews

The interviewer (MT) started the interviews by stating the study objectives and research questions; thereafter, she clarified what was meant by ‘endoscopy pathway’ (Figure 2).

### 2.4 Data processing/Data analysis

**FIGURE 3:**
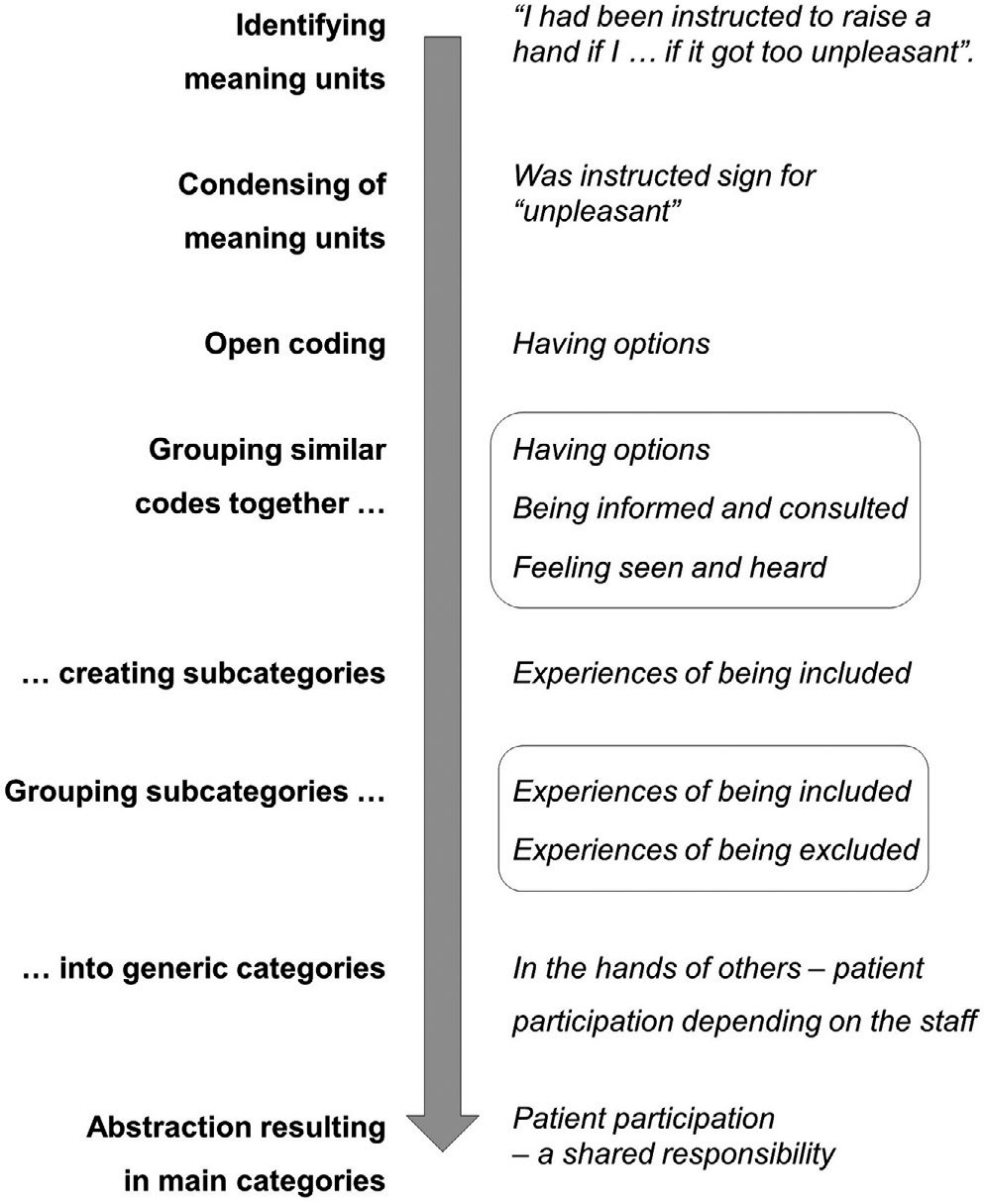
Schematic figure of qualitative analysis process

The methodological orientation of this qualitative interview study was an inductive content analyses, as described by Elo and Kyngäs. All interviews were audiorecorded and transcribed verbatim. The transcripts were thoroughly read and reflected upon, both individually and later during workshop meetings. Analysis followed by identifying units of meaning in the transcripts. These were condensed, and open coding was used. Subcategories were grouped, which in turn formed generic categories, and were finally grouped in two main categories. The coding was performed by the first (HD) and last (MB) authors, partly together and partly separately as a form of triangulation. To avoid fragmentation and loss of meaning, the interviewer validated the analysis in relation to her experience from the interviews. The codes and categories were translated to English after completion of the analysis, to avoid information being lost in translation. This process is described in Figure 3. Software used for the data managing was Excel in Microsoft Office 16.

### 2.5 Ethics

The Regional ethics committee in Stockholm approved the study: Dnr 2017/1341‐31/2. All results are presented in such manners that none of the participants nor health‐care personnel can be identified. During the study period, the first author (HD) was employed at the endoscopy unit from which the participants were recruited. Participants were informed in writing and orally that participation was voluntary. All participants consented to the study in writing prior to the interviews.

## 3 RESULTS

Thirty‐four patients consented to their contact information being transmitted to the interviewer. Seventeen patients consented to participate in the interviews, and the remainder were either not possible to get a hold of or withdrew their interest due to time constraints. The interviews lasted 12‐45 minutes (median 22 minutes). Informant characteristics are presented in Table 1.

**TABLE 1:** Characteristics based on the study informants (n = 17)

	n = 17
Age, median (IQR)	68 (52‐72,5)
Age, total range	19‐80
Gender	
Female	8
Male	9
Education level	
Compulsory school (<9 y)	3
Upper secondary school (9‐12 y)	6
University (>12 y)	8
Procedure	
Gastroscopy (diagnostic and/or therapeutic) including endoscopic ultrasonography	10
Colonoscopy (diagnostic and/or therapeutic)	7
Swedish as first language	12

The inductive analysis resulted in five generic categories: (a) *subject or object—the patient's role as a participant*, (b) *in the hands of others—patient participation depending on the staff*, (c) *organizational factors influence possibilities for participation*, (d) *impressions of staff set the tone for participation* and (d) *individual circumstances affect readiness for participation*. The generic categories were grouped in two main categories: (a) *patient participation—a shared responsibility*, and (b) *vulnerability to external and internal factors* (Table 2).

**TABLE 2:** Descriptions of main categories, generic categories and subcategories

Main categories	Generic categories	Subcategories
Patient participation—a shared responsibility	Subject or object—the patient’s role as a participant	Active participation
		Passive participation
	In the hands of others—patient participation depending on the staff	Experiences of being included
		Experiences of being excluded
Vulnerability to external and internal factors	Organizational factors influence possibilities for participation	Experiences of getting an appointment
		Importance of the physical environment
		Accessibility
	Impressions of staff set the tone for participation	Perceptions of the staff
	Individual circumstances affect readiness for participation	Personal circumstances
		Drug effects on body and cognition

### 3.1 Patient participation—a shared responsibility

Informants' descriptions of their experiences and attitudes on their involvement in endoscopy could be labelled as either active participation or passive participation, and sometimes both approaches were described by the same informant. These accounts included both the patients' own behaviour and attitudes as well as in response to the staff's actions. The descriptions could be separated in two groups: the patient's own role and the professional's position of power.

#### 3.1.1 Subject or object—the patient's own role as a participant

##### Active participation

Reports on participation that were labelled as active mostly regarded information seeking and/or information transmission. The informants had searched the internet, talked to friends and called the unit to get informed or to discuss medication.

I read carefully. If I don't understand, I make a phone call and ask immediately, so I get an answer. #13

Active participation also included expressing preferences to the endoscopy staff on sedation and location for the intravenous catheter. One informant had negative experiences of a certain kind of laxative for bowel preparation. When he had gotten a new prescription of the same kind of laxative, he called the unit to ask for another kind. The nurse arranged for another prescription according to his preference.

##### Passive participation

A common perception among the informants was that the endoscopic examination was something ‘you just needed to go through’ without having influence or even opinions on how it should be done. These descriptions often seemed based on the assumption that the health‐care professionals knew what was best for the patient. Some of the informants stated that they did not know the exact reason for their endoscopy.

One of the older informants described a cautiousness in the interaction with the professionals.

I don't know how much you should interfere, I mean as a patient. I don't think you can have an influence, it's difficult. You shouldn't be too cocky. […] you need to be careful […] you are a patient and that's just the way it is. #3

#### 3.1.2 In the hands of others—patient participation depending on the staff

Staff was described to be in a position of power; thus, staff's actions were vital to the informants' experiences of patient participation.

##### Experience of being included

The sense of being included could occur throughout the contact with the unit. Information preceding the endoscopy was of significance for the feeling of preparedness before the procedure. Information leaflets in the notice to attend the appointment and staff explaining to the patient during the visit were mentioned as helpful.

The informants described that options given to them at the appointment mainly regarded the possibility of sedation. When staff continuously informed the patient how the examination went on and made sure that the patient was feeling well by asking questions, the informants felt at ease and involved in their endoscopy.

It was all positive, and the doctor explained it well to me. I got to watch the screen and everything. #14

A few informants said they had been given the option to stop the examination if needed. One informant had been instructed to raise a hand during the gastroscopy if it became too unpleasant. Several of the informants expressed a feeling of being acknowledged and had the impression of an 'open atmosphere’.

##### Experience of being excluded

The informants wished for information to be prompt, clear, upfront and reinforced in writing. When staff had failed to meet information needs, the informants expressed feelings of anxiety and uncertainty, especially when it came to results of the endoscopy. As a result of insufficient information, patients could make conclusions based on interpretations of the staff's actions or behaviour.

But apparently, it's nothing dangerous since everyone is calm, but I don't know what it is. So, I have to trust the fact that no one else is reacting, so one has to hope that it's all good. #2

Some of the informants described feelings of being denied opportunities of active participation. One such example was when the patient had not been given the chance to discuss options for treatment or investigation, neither with the referring physician nor the endoscopist. Some informants had the impression that staff withheld information, especially when it came to results of the examination.

The only thing [the endoscopist] said was that I did good, since I didn't find it painful. It didn't hurt because I slept the whole time. [The endoscopist] didn't say if it went well or not. #7

The interviews included descriptions of the exposed and vulnerable position of patients undergoing endoscopy. One informant described that the endoscopist would not administer more analgesics and did not explain why, although the colonoscopy was painful. There were accounts on staff behaviour leading to feelings of being neglected, such as staff talking to one another during the examination without addressing the patient.

Well, someone came in and talked to the nurses. I suppose that's the way it has to be. I think, it's the feeling of not being … if somebody would have come through the door […] normally I would have gotten glanced at and the person would say something. But in this situation, we're talking about, I get reduced from a patient to an object. Well, that's not so fun. Maybe it is understandable, but it's not fun. #6

### 3.2 Vulnerability to external and internal factors

Many factors that had an influence on the endoscopy experience also impacted patient participation. Three groups of such factors were identified: organizational, staff‐related and patient‐related.

#### 3.2.1 Organizational factors influence possibilities for participation 

The informants brought up topics that were labelled as organizational factors. These affected the perception of having access to the unit in different ways and was something that patients themselves could not influence.

##### Experiences of getting an appointment

Reports on appointment logistics involved descriptions of waiting for the notice to attend and the booking of appointment. A reoccurring issue in the interviews was the feeling of uncertainty while waiting for the notice.

And then nothing happened, we didn't get an answer or anything. I didn't even know if they had received the referral. #15

Preferences on booking the appointment varied. Some informants wished to use an online booking system, while others were satisfied with a notice by postal delivery. Despite the differences in opinions, the appointment booking logistics appeared to be of major importance to the informants.

##### Importance of the physical environment

The environment was often mentioned by the informants, in particular the waiting room area, which was perceived as unwelcoming. Some of the informants stated that they had wished for health‐care professional presence in the waiting room. Other environment‐related topics included experienced difficulties to find the way to the unit while at the hospital.

##### Accessibility

Patients' own electronic access to health‐care records was not yet introduced, which was something a few of the informants requested.

Some informants expressed a desire for an increased accessibility to the endoscopy staff. Getting through to the unit by telephone was described as difficult, especially for urgent matters, such as being late to an appointment. One informant had a suggestion for access to the staff after the examination.

You could also have a phone number, preferably a direct line to the nurse or the doctor who were there [during the examination]. #11

#### 3.2.2 Impressions of staff set the tone for participation

##### Perceptions of the staff

The informants described their impressions of the endoscopy staff, in words such as welcoming, kind, informative and stressed. Opinions on the endoscopy professionals' individual competencies were expressed by some of the informants, in a positive, negative or neutral tone.

I got the impression that [the nurse] was used to stressed and anxious patients, since this examination isn't exactly the most popular one. #12

The punctuality of the staff was also a reoccurring topic in the interviews. Either the endoscopy starting right on time or having to wait mattered.

As both staff at the study unit and many of the patients visiting the unit were of different cultural origin, language was sometimes mentioned as a barrier for the communication; however, this was stated to be of minor importance to those mentioning it.

#### 3.2.3 Individual circumstances affect readiness for participation

Descriptions related to the patient, either their own history, social context, feelings or reactions but also individual reactions to drugs were grouped and labelled as individual circumstances.

##### Personal circumstances

The informants described multiple personal circumstances that could influence their active participation. Expectations or fears were often related to previous experiences and understanding of the procedure, and these in turn could influence the ability to assimilate information and to take an active part in decision making. The personal motivation to undergo the examination was of importance in accepting the procedure although it was perceived as unpleasant.

There is a certain discomfort, you could say … absolutely … But you do it because you have to, so there won't be any harm. #17

Negative feelings, such as anxiety or panic, in relation to the procedure were described by some participants, while others expressed an acceptance of the physical discomfort. Meditation and calm breathing were examples of strategies used for coping. The next of kin accompanying one of the younger informants had an important role for maintaining a sense of control.

I knew that [next of kin] would speak up if something was bad or so #15

##### Drug effects on body and cognition

Descriptions regarding effects of the bowel preparation were expressed in negative terms. It often led to weariness and fear of incontinence on the way to the hospital. Sedative drugs caused substantial amnesia for some of the informants. Comprehending oral information after the procedure was described as difficult under the effect of sedation.

Maybe I was a little dizzy, so afterwards, I didn't understand. [The endoscopist] quickly drew on a piece of paper, like that … but where in the body that is, I don't have a clue. #6

To illustrate the findings in this study, examples and possibilities for patient participation, derived from the interviews, are added to a timeline over the endoscopy pathway (Figure 2).

## 4 DISCUSSION

In this study, in which patient experiences of patient participation in endoscopy were explored, a picture of shared responsibility between the patient and the health‐care professionals emerged. Multiple factors that influenced patient participation in this specific context were identified.

Although several examples of active patient participation and factors contributing thereto, such as self‐care and symptom management, trust in the professionals, coping behaviours and future adherence to screening have been described in endoscopy patient experience research,^23^, ^26^, ^30^ to the best of our knowledge, this is the first qualitative patient interview study in GI endoscopy, with the explicit objective to explore patient participation.

Our aim was to gain knowledge on patients' experiences, attitudes and preferences concerning patient participation in endoscopy. We found descriptions of experiences, attitudes and preferences of importance for patient participation in each step of the endoscopy pathway. The findings contribute to the literature on endoscopy patient experiences and on patient participation in short‐term clinical encounters.

Informants described many approaches to their care that were interpreted as either active or passive. Reports on behaviour or actions that were interpreted as illustrative to an active patient role mainly concerned information seeking and information transmission. Our interpretation of the active patient role, described by the informants, corresponds to the most basic form of engagement (the patient is merely informed) in the ‘level of direct care’ in Carman et al's framework.^7^ Information to the patient, weather it was written or oral, was important for the perception of having been included or excluded in the endoscopy. Nevertheless, there were some examples of a more advanced degree of engagement in the interaction between staff and patients. This occurred when staff ‘invited’ patients to participate in decision‐making concerning sedation or when offering a possibility to discontinue the examination. Thus, the informants felt acknowledged and involved. This is in line with previous research findings, where endoscopy nurses have an important role for patients to support them in maintaining control and managing anxiety.^24^

In Cahill's concept analysis, patient participation could be achieved when the health‐care professional surrendered some of his/her power or control to the patient.^11^ Patient participation cannot occur without the willingness of the patient and the professional. Understanding and acknowledging patients' individual circumstances are key factors to support active patient participation.^11^, ^31^ This requires an open dialogue with information exchange between the patient and the health‐care professionals. In our study, we however found examples of the complete opposite. Many informants described a passive approach to their care, which can be related to the traditional ‘paternalistic’ model, where the patient is a passive recipient of care and the professional is the expert.^32^ Some of the informants did not know, even at the point of the interview, the exact reason for their endoscopy. They did what they were told, without knowing why—a behaviour which may be interpreted as the ultimate submission in a paternalistic patient‐professional relationship.

Eldh et al^10^ described *non‐participation* in a Swedish health‐care context, which was divided into three domains: lack of knowledge, lack of respect and passiveness. In our study, we found that several of the informants ‘handed over their body for a check‐up’ when going through endoscopy, as if their body and mind were temporarily separated. This attitude was similar to the passiveness described by Eldh et al and was not necessarily further reflected upon by the informants. Some informants experienced that staff ‘withheld information’, ‘talked over their heads’ and ‘reduced them from a person to an object’. These accounts resemble Eldh et al's descriptions of lack of respect. Excluding behaviour from the staff's part, although possibly unintentional, together with the factors described above (eg effects of sedation) hinder patient participation.

Many of the descriptions of patient participation occurred in the interaction between the patient and the professional. However, as Carman et al point out, in ‘the level of direct care’, also other actions outside the clinical setting can stimulate active patient engagement. In this study, such factors were found within organizational, staff‐related and patient‐related areas. Among the organizational factors, the importance of the waiting room area was unexpected to us. Endoscopy has been associated with moderate‐to‐severe anxiety in relation to the preparations, the examination itself and the fear of illness.^13^ It might be that the waiting room is the location where all built up expectations and anxiety peak. In our study, it seemed to represent something more than just the first impression of the physical environment. The sense of trust or distrust in the care providers might start earlier than one would expect.

Accessibility to medical records and an open phone line to the unit were subjects of importance to the informants. Without access to information and dialogue with staff, patient participation is difficult to achieve. Carman et al described access to medical records to illustrate the degrees of engagement: When patients only can access their medical records through the clinicians, the degree of engagement is low. When patients may read their online medical records, engagement reaches the midpoint degree. Finally, when patients can edit or add information in their medical records, the highest degree—the partnership has been reached.^7^

Although some factors of importance for patient participation were strictly related to the patient (eg previous experiences, support from family), most factors, such as punctuality and appointment logistics, could be influenced by the health‐care professionals or by the health‐care organization.

Our findings suggest that patients' experience an inferior position in the interaction with endoscopy staff. In a systematic literature review, Angel and Frederiksen described that health‐care personnel's attitudes on patient participation are of great importance, but also that sufficient time is needed to build a relationship where patient participation can take place.^33^ It is likely that the time factor is more of a challenge in endoscopy than many other care settings (eg geriatric home care, rehabilitation). Endoscopy is often a high‐volume service in which sometimes a relationship must be established in just a few minutes before starting a procedure. Many endoscopy units struggle with the difficult equation of having to ‘produce care’ in a limited time while still maintaining high quality. If patient participation in endoscopy is valued, time to enable patient participation needs to be prioritized.

The impressions of staff were frequently mentioned by the informants. They made assumptions on staff professionalism and state of mind (eg stressed) based on overt behaviour and communication. In Cahill's definition of patient participation, the relationship between the patient and the professional is essential^11^ and interpersonal communication skills have been identified as supportive for patient participation.^31^ Therefore, impressions of how staff demonstrates accessibility, attention and hospitality can be interpreted as a fundament on which expectations and trust for coming interactions are built.

The (often desired) dizziness and amnesia evoked by the sedation agents affect the processing of information and ability to take an active role. This matter always needs to be taken into consideration, even when patients appear to be fully alert. Many patients need time to recover from the sedation to understand information given to them, ask questions and assume an active participation.

An important gain connected to patient‐centred care, at times not acknowledged, is increased patient safety. An active and vigilant patient can serve as safety barrier in preventing adverse events.^32^, ^34^ In gastrointestinal endoscopy, risks include patient misidentification, negative drug effects such as allergic reactions, sedation‐related complications, cardiopulmonary problems, haemorrhage and perforation.^35^, ^36^, ^37^ Knowledge about the patient's health status and medical history is crucial for reducing some of these risks. Such information is sometimes only accessible to the professionals through a dialogue with the patient. Therefore, patient participation in GI endoscopy should be seen also as promoting patient safety and given at least the same amount of attention as other important safety measures.

Finally, it is important to understand how policies on patient participation are translated into practice. Therefore, future studies should also focus on endoscopy professionals' attitudes to patient participation and experiences on promoting patient participation to understand the interpersonal dynamics in this matter.

### 4.1 Strengths and limitations

The research group was multiprofessional with experience from different medical fields, such as oncology, gastroenterology and anaesthesiology. This diversity in the group enriched our research discussions and analyses. The participants were a heterogenous group, differing in age, sex, educational‐ and cultural backgrounds, hence reflecting our practice.

However, our study has limitations. The interview guide was developed within the research group, without influence from endoscopy patients, which could have been valuable.

All participants were fluent in Swedish, and only outpatients were included in our sample, which is a limitation to the transferability of our results. This should be taken in consideration when interpreting our study results, as the perspectives on patient participation of non‐Swedish speakers and inpatients could differ from our sample's descriptions.

Although the interviews were conducted by a nurse who was not involved in the patients' care, she was employed at the hospital's other endoscopy unit. This could potentially have influenced the participants in their interviews. They could, on one hand, have wanted to answer ‘desirably’, and on the other hand, they had an opportunity to tell their story to someone who was familiar with the endoscopy context.

## 5 CONCLUSIONS

In this study, endoscopy patients' descriptions of their experiences, attitudes and preferences on patient participation throughout the endoscopy pathway have been explored. A picture of a joint responsibility of patient participation between professionals and patients in endoscopy emerged. The balance of power is however not equal, as the professionals are in a superior position and lack of time precludes attempts to level this. Patient participation is vulnerable to both patient‐related factors and external factors. In conclusion, endoscopy patients need support from professionals to participate actively. We have identified several ways to actively involve patients, acknowledge personal circumstances and be aware of factors that may influence patient participation throughout the endoscopy pathway.

## CONFLICT OF INTEREST

The authors declare no conflict of interest.

## Data Availability

The data that support the findings of this study are available on request from the corresponding author. The data are not publicly available due to privacy or ethical restrictions.
